# Cell-Free Fat Extract Prevents Tail Suspension–Induced Bone Loss by Inhibiting Osteocyte Apoptosis

**DOI:** 10.3389/fbioe.2022.818572

**Published:** 2022-01-28

**Authors:** Mingming Xu, Jingke Du, Junqi Cui, Shuangyan Zhang, Shuhong Zhang, Mingwu Deng, Wenjie Zhang, Hanjun Li, Zhifeng Yu

**Affiliations:** ^1^ Shanghai Key Laboratory of Orthopedic Implants, Department of Orthopedic Surgery, Shanghai Ninth Peoples Hospital, Shanghai Jiao Tong University School of Medicine, Shanghai, China; ^2^ Knee Surgery Department of the Institute of Sports Medicine, Beijing Key Laboratory of Sports Injuries, Peking University Third Hospital, Beijing, China; ^3^ Department of Pathology, Shanghai Ninth Peoples Hospital, Shanghai Jiao Tong University School of Medicine, Shanghai, China; ^4^ Shanghai Key Laboratory of Tissue Engineering, Department of Plastic and Reconstructive Surgery, Shanghai Ninth People's Hospital, Shanghai Jiao Tong University School of Medicine, Shanghai, China; ^5^ Clinical Stem Cell Research Center, Ren Ji Hospital, Shanghai Jiao Tong University School of Medicine, Shanghai, China

**Keywords:** cell-free fat extract, hind limb, osteoporosis, osteocyte, apoptosis, lacunar-canalicular system, ERK signaling pathway

## Abstract

**Introduction:** As the space field has developed and our population ages, people engaged in space travel and those on prolonged bed rest are at increasing risk for bone loss and fractures. Disuse osteoporosis occurs frequently in these instances, for which the currently available anti-osteoporosis agents are far from satisfactory and have undesirable side effects. CEFFE is a cell-free fraction isolated from nanofat that is enriched with a variety of growth factors, and we aim to investigate its potential therapeutic effects on disuse osteoporosis.

**Methods:** A tail suspension–induced osteoporosis model was applied in this study. Three weeks after tail suspension, CEFFE was intraperitoneally injected, and PBS was used as a control. The trabecular and cortical bone microstructures of the tibia in each group were assessed by μCT after 4 weeks of administration. Osteocyte lacunar-canalicularity was observed by HE and silver staining. *In vitro*, MLO-Y4 cell apoptosis was induced by reactive oxygen species (ROSUP). TUNEL staining and flow cytometry were used to detect apoptosis. CCK-8 was used to detect cell proliferation, and Western blotting was used to detect MAPK signaling pathway changes.

**Results:** CEFFE increased the bone volume (BV/TV) and trabecular number (Tb.N) of the trabecular bone and increased the thickness of the cortical bone. HE and silver staining results showed that CEFFE reduced the number of empty lacunae and improved the lacuna-canalicular structure. CEFFE promoted osteocyte proliferative capacity in a dose-dependent manner. CEFFE protected MLO-Y4 from apoptosis by activating the serine/threonine-selective protein kinase (ERK) signaling pathways.

**Conclusion:** CEFFE attenuated immobilization-induced bone loss by decreasing osteocyte apoptosis. CEFFE increased the survival of osteocytes and inhibited osteocyte apoptosis by activating the ERK signaling pathway *in vitro*.

## Introduction

Osteoporosis (OP) is a metabolic bone disease in which bone mass is lost and the bone organic matrix and bone minerals are reduced (Lane et al., 2000), and it is mainly manifested as low bone mineral density and bone tissue structure destruction, which easily leads to fracture with the evolution of the disease (Conference, 2001). Long-term bed rest caused by trauma or spinal cord injury is a major cause of disuse osteoporosis (Bloomfield, 1997), and with the development of the space industry worldwide in recent years, astronaut’s bone loss caused by a microgravity environment has gradually attracted attention (LeBlanc et al., 2000). Osteocytes are mechanosensitive cells and can sense mechanical changes in the environment (Klein-Nulend et al., 2012; Klein-Nulend et al., 2013); thus, in paralyzed people and astronauts, hypodynamic situations reduce the mechanical stimuli received by osteocytes and cause abnormalities in bone antiresorptive and bone metabolic activities.

In response to bone loss caused by long-term bed rest and weight loss, previous studies have found that bisphosphonates and traditional antiresorptive agents do not work well in disuse osteoporosis (Li et al., 2004). Therefore, an increasing number of studies have focused on osteocytes to rescue bone loss. Histone deacetylase 5 (HDAC5) in bone marrow mesenchymal stem cells play an important role in controlling bone remodeling. Recent studies used the tetrahedral nucleic acids framework (tFNAs) to transport miR-2861, which could inhibit HDAC5 expression, promoting osteogenic differentiation (Li et al., 2021a; Zhang et al., 2021). Sclerostin, an inhibitor of the Wnt/β-catenin signaling pathway that regulates bone growth, has emerged as an attractive therapeutic target for the treatment of osteoporosis (Suen and Qin, 2016). Dongye Zhang et al. used the sclerostin antibody to retain osteocytic micromorphology and function to rescue bone mass against prolonged mechanical unloading (Zhang et al., 2017; Zhang et al., 2020), while a phase 3 trial reported that patients administered with romosozumab (sclerostin monoclonal antibody) may experience some adverse events, such as nasopharyngitis, arthralgia, and hypercalcemia (Langdahl et al., 2017). Yi-Xian Qin et al. showed that low-intensity, high-frequency loading has the potential to mitigate regional bone loss induced by long-term bed rest (Qin et al., 2019). Likewise, J. Sibonga et al. found that advanced resistive exercise could attenuate bone mineral density defects caused by weightlessness but could not suppress elevated resorption biomarkers (Sibonga et al., 2019). Thus, seeking a better therapeutic strategy for disuse osteoporosis is still a continuously explored process.

Cell-free fat extract (CEFFE) was first described in our previous study (Yu et al., 2018; Xu et al., 2020). CEFFE is extracted from human adipose tissue, which seems to be the most convenient tissue for human separation because of its subcutaneous location, lower amount of trauma caused to the human body, and the need for minimally invasive techniques for the operator. Furthermore, CEFFE is a cell-free liquid that greatly reduces its immunogenicity and ensures its safety during treatment. Our previous studies have found it to have pro-angiogenic activity (Yu et al., 2018) and found that it improved skin flap survival (Cai et al., 2019), improved fat graft survival (Zheng et al., 2019), and promoted the healing of diabetic wounds (Wang et al., 2020; Yin et al., 2020). CEFFE contains cytokines and growth factors such as IGF1, bFGF, and other growth factors, which can promote bone growth (Pedersen and Febbraio, 2012). Therefore, it is worth exploring its therapeutic effect in osteoporosis.

The objective of this study was to evaluate the therapeutic effects of CEFFE in mitigating disuse bone loss in a tail suspension mouse model. Moreover, to investigate the underlying mechanisms by which CEFFE rescues bone mass, apoptosis-related proteins and matrix-degrading proteins were examined, and the ability of CEFFE to inhibit osteocyte apoptosis was evaluated in MLO-Y4 cells *in vitro*.

## Materials and Methods

### CEFFE Preparation

CEFFE was provided by Shanghai Stem Cell Technology Co., Ltd. (Shanghai, China). The extraction of CEFFE was performed as described previously (Qin et al., 2019). In brief, the fresh fat obtained from healthy volunteers was mechanically emulsified after centrifugation and the third aqueous layer is retained after re-centrifugation, filtered using a 0.22-μm filter, and stored at −80 °C. The CEFFE protein concentration was 5,000 μg/ml detected using a bicinchoninic acid assay kit (Beyotime Biological Technology Institution, Shanghai, China).

### Animals

The animal operation procedures were approved by the Committee of Ethics on Animal Experiments at the Shanghai Jiao Tong University School of Medicine. Eight-week-old C57BL/6 male mice (Shanghai SIPPR BK Laboratory Animals Ltd., Shanghai, China) were housed individually in a temperature-controlled animal facility with a 12-h light/dark cycle and free access to chow diet and water.

### Tail Suspension Mice Model

Each mouse’s tail was taped to a rope, and the mice were suspended through a pulley system on the top of a customized cage. The mice could walk in the cage on their forelimbs, which remained in contact with the cage floor, while their hind limbs remained suspended with their body at a 30° head-down angle to mimic microgravity (Plotkin et al., 2005). Food and water were provided on the cage floor.

Eighteen mice were randomly divided into three groups (*n* = 6/group): a control group with normal gravity (Normal), a tail-suspended group that was injected intraperitoneal (i.p.) with 250 μl of PBS as vehicle (TS + vehicle), and a tail-suspended group that was injected i.p. with 250 μl [the administration dose of CEFFE was referred to our previous published article (Cai et al., 2019)] of CEFFE (TS + CEFFE) twice a week for 4 weeks after suspension for 3 weeks, and the mice were still in tail suspension during the administration period. The extraction of CEFFE was performed as described previously (Yu et al., 2018). The mice were sacrificed by cervical dislocation after anesthetization with pentobarbital. Left and right tibias were isolated.

### Microcomputed Tomography Analysis

Right tibias were scanned by a high-resolution μCT scanner (μCT 80; Scanco, Zurich, Switzerland) to obtain the trabecular and cortical bone microstructure. Tshe scanning parameters were set as follows: voltage, 70 kV; electric current, 114 μA; and resolution, 10 μm per voxel. For trabecular measurements, a region of interest was defined at 1.9 mm from the proximal tibial condyles, immediately distal to the growth plate, and extended to 100 slices. For cortical bone analyses, a region of interest was defined at mid-diaphysis, starting 4.5 mm from the proximal tibial condyles and extended to 100 slices. The microarchitecture parameters included bone volume fraction (BV/TV, %), trabecular number (Tb.N, 1/mm), trabecular separation (Tb.Sp, mm), connection density (Conn.Dens, 1/mm³), and cortical bone thickness (Ct.Th).

### Histomorphological Analysis of the Lacunar-Canalicular System

The left hind limbs were fixed with 4% paraformaldehyde (PFA) for 24 h, followed by running water for 4–8 h, transferred to 10% EDTA (pH 7.4), and placed in a 4 ºC refrigerator for decalcification for 28 days. Bone samples were embedded in paraffin and sectioned (5 μm thick). Tissue sections were stained with hematoxylin and eosin (H&E). Images were obtained at 20x and 40x magnification for analysis, and the proportion of empty bone lacunae in each group was counted.

Silver staining was performed as previously reported (Feng et al., 2020). In brief, the sections were deparaffinized and incubated for 55 min in a gelatin solution containing two parts 50% silver nitrate and one part 1% formic acid. Stained slides were then washed in 5% sodium thiosulfate for 10 min and subsequently dehydrated, clarified, and fixed. Consistent cortical regions were selected for assessment in the medial and lateral regions of the femur midshaft in each specimen. Images were obtained at 100x magnification for analysis. In the cortical bone regions, canalicular length was quantified using ImageJ (NIH, Bethesda, and Maryland) (Java 1.8.9_66).

### Immunohistochemistry Stain

After deparaffinization and hydration with distilled water, the antigens were retrieved with 0.25% trypsin, and the peroxidase was inactivated (3% H_2_O_2_). Sections were incubated overnight at 4°C with primary antibodies against cleaved caspase-3 (1:1,000, CST) and matrix metalloproteinase-13 (MMP13) (1:1,000, CST), followed by horseradish peroxidase–conjugated secondary antibody. Peroxidase was reacted with 3,3′-diaminobenzidine. Consistent cortical bone regions were selected in the medial and lateral regions of the femur midshaft in each specimen. Total osteocytes and positively stained cleaved caspase-3 and MMP-13 osteocytes were counted.

### Cell Culture

MLO-Y4 cells were generously provided by Dr. Lynda F. Bonewald. MLO-Y4 cells were cultured in *a*-MEM with 5% fetal bovine serum (FBS), 5% calf bovine serum (CBS), and 50 μg/ml gentamicin sulfate under a humidified atmosphere (37 °C, 5% CO_2_). Cells were seeded in culture dishes preplated with rat tail type I collagen (Corning Inc., Corning, NY, United States), the medium was replaced every 2 days, and cell passaging was performed when the monolayer of adherent cells reached 80–90% confluence. An osteocyte apoptosis model was established with Rosup (50 μg/ml), and cells were treated with CEFFE (250 μg/ml).

### Cell Proliferation Assays

MLO-Y4 cells were seeded in a 96-well plate at 8 × 10^3^ cells per well and maintained in a complete medium. After 12 h, the cells were incubated with different concentrations of CEFFE (0, 25, 50, 100, 250, and 500 ng/ml) for 24 h. A cell Counting Kit-8 (CCK-8; Weiao Biotechnology, Shanghai, China) was used to evaluate cell proliferation. The absorbance spectrum at 450 nm was recorded using a microplate reader, and the absorption spectrum at 620 nm was recorded as the reference wavelength (SpectraMAX i3x; Molecular Devices, Sunnyvale, CA, United States). The data are presented as the ratio of the O.D. value relative to the control group without CEFFE.

### Flow Cytometry

MLO-Y4 cells were cocultured with different concentrations of CEFFE (50, 250, and 500 μg/ml), and cell cycle analysis was performed after 24 h. Cultured cells were collected and fixed with 70% ethanol overnight, followed by incubation with RNase A (Beyotime Biological Technology Institution, Shanghai, China) and propidium iodide (Beckman-Coulter, Brea, CA, United States).

Apoptosis of MLO-Y4 cells induced by Rosup (a compound mixture with 4-butylhydroperoxide included) was determined by flow cytometry using the Annexin-V/PI Apoptosis Detection Kit (Becton Dickinson and Co., Franklin Lakes, NJ, United States). In brief, MLO-Y4 cells were stimulated for apoptosis with Rosup, and the experimental group was incubated with CEFFE (250 μg/ml) for 8 h. In subsequent experiments to examine the mechanism of CEFFE anti-apoptosis, ERK, p38, and JNK inhibitors (10 μM), that is, GDC-0994, SB203580, and PD98059, were added to the cells incubated with Rosup and CEFFE for 8 h. Then the cells were harvested, washed twice with cold PBS, and labeled with FITC Annexin V and PI in a binding buffer. The cells were then submitted to flow cytometry using a BD LSR Fortessa system (Becton Dickinson and Co.) to detect the fluorescence intensity of the cells. The experiment was repeated three times, and the apoptosis rate (%) of each group was calculated.

### RNA Extraction and qRT-PCR

Total RNA from cells was extracted using the TRIzol reagent (Thermo Fisher Scientific, 15596026), according to the manufacturer’s instructions. Reverse transcription was performed using a Biomake Supermix Kit (Bimake, Houston TX, United States). Diluted complementary cDNA was analyzed by qPCR using the SYBR Green reagent (Bimake). Quantitative real-time PCR (qRT-PCR) primers used in the study were designed using PrimerBlast (https://www.ncbi.nlm.nih.gov/tools/primer-blast/) and are listed in Table 1.

**TABLE 1:** Primers used in real-time PCR.

Gene	Forward primer	Reverse primer
Gapdh	ATG​GTG​AAG​GTC​GGT​GTG​AA	TGA​GTG​GAG​TCA​TAC​TGG​AAC​A
MMP13	TTT​CTT​TAT​GGT​CCA​GGC​GAT​GA	AGG​CGC​CAG​AAG​AAT​CTG​TC
Sost	AGG​CGC​CAG​AAG​AAT​CTG​TC	AGG​CGC​CAG​AAG​AAT​CTG​TC
Rankl	CAG​CAT​CGC​TCT​GTT​CCT​GTA	CTG​CGT​TTT​CAT​GGA​GTC​TCA

### TUNEL Assays

TUNEL assays were performed using a One-Step TUNEL Apoptosis Assay Kit (Beyotime Biological Technology Institution, Shanghai, China) to detect apoptotic cells. In brief, cells were fixed with 4% PFA for 30 min. PBS containing 0.3% Triton X-100 was added and incubated for 5 min at room temperature. Fifty microliters of TUNEL detection solution was then added to the samples and incubated for 60 min at 37 °C in the dark. The slides were mounted with anti-fluorescence quenching mounting solution and observed under a confocal fluorescence microscope. The excitation wavelength range was 450–500 nm, and the emission wavelength range was 515–565 nm (green fluorescence).

### Western Blotting

MLO-Y4 cells were seeded in collagen-coated 12-well plates at 2.0 × 10^5^ cells per well, maintained in *a*-MEM containing 0.5% FBS and 0.5% CBS for 3 h, and then transferred into a medium containing 5% FBS, 5% CBS, and CEFFE (250 μg/ml). Total protein was collected from each group of MLO-Y4 cells at different time points (0, 5, 10, 15, 30, and 60 min) after changing the medium. Proteins were cleaved with the SDS lysis buffer (Beyotime Biological Technology Institution), extracted, subjected to SDS–polyacrylamide gel electrophoresis (SDS-PAGE, 15%), and then transferred to polyvinylidene difluoride (PVDF) membranes. Incubation with the primary antibodies anti-pERK, anti-Erk, anti-p38, anti-p-p38, and β-actin was performed overnight at 4 ºC. Subsequently, the membranes were incubated with anti-rabbit IgG (CST, Danvers, MA, United States) for 1 h at room temperature. Finally, protein bands were visualized with an Odyssey infrared imaging system (LI-COR Biosciences, Lincoln, NE, United States).

### Statistical Analysis

The results are expressed as mean ± SD. All data were analyzed with GraphPad Prism 9 (GraphPad Software, United States), and differences were analyzed by one‐way ANOVA, followed by Tukey’s post hoc test (group >2). All tests were performed with significance levels of *p* < 0.05 and *p* < 0.001.

## Results

### CEFFE Improved Bone Mass in Osteoporotic Mice Induced by the Tail Suspension Model

**FIGURE 1:**
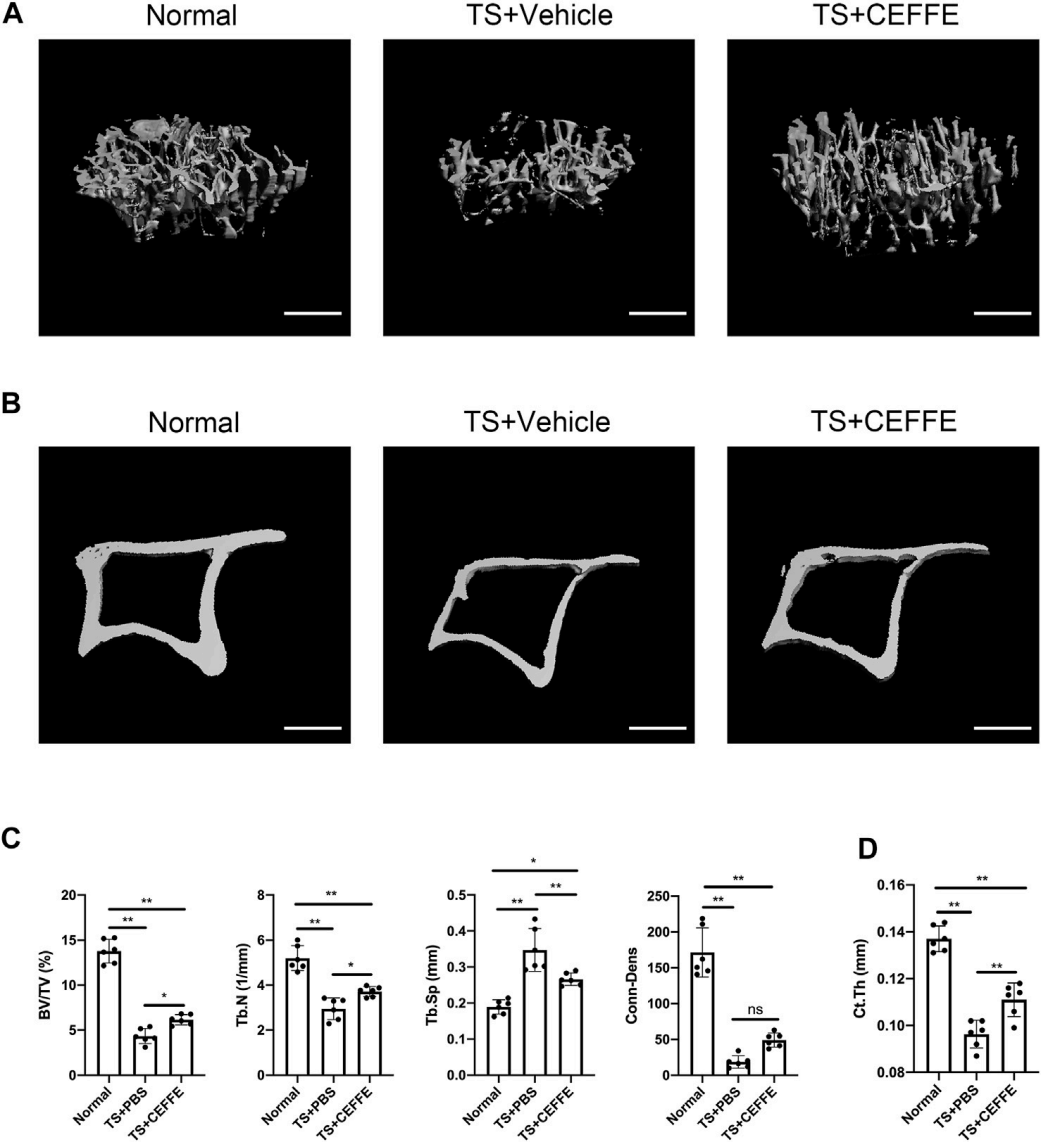
CEFFE improved bone mass in osteoporotic mice induced by the tail suspension model. Three-dimensional (3D) reconstruction from microcomputed tomography (µC+T) scans of proximal tibia from normal mice, mice submitted to tail suspension + vehicle, and mice submitted to tail-suspension + CEFFE **(A)**. Representative μCT images of the cortical bone **(B)**. Quantitative analyses of the trabecular bone of the proximal tibia **(C)**, and the cortical bone thickness in all groups **(D)**. Scale bar, 500 μm *p < 0.05, and **p < 0.01.

As shown in Figures 1A,C, tail suspension caused significant trabecular bone loss compared to wild-type mice. The trabecular bone impairment was ameliorated in the TS + CEFFE group, with increased BV/TV (%), Tb.N (1/mm), and Conn.Dens levels compared with the TS + vehicle group, but Conn.Dens was not different between the TS + CEFFE and TS + vehicle group. Meanwhile, the Tb.Sp (mm) was apparently decreased (Figure 1B). CEFFE also increases the cortical bone thickness of the tibia after tail suspension (Figures 1B,D).

### CEFFE Improved the Lacunocanalicular Microstructure in Tail-Suspended Mice

**FIGURE 2:**
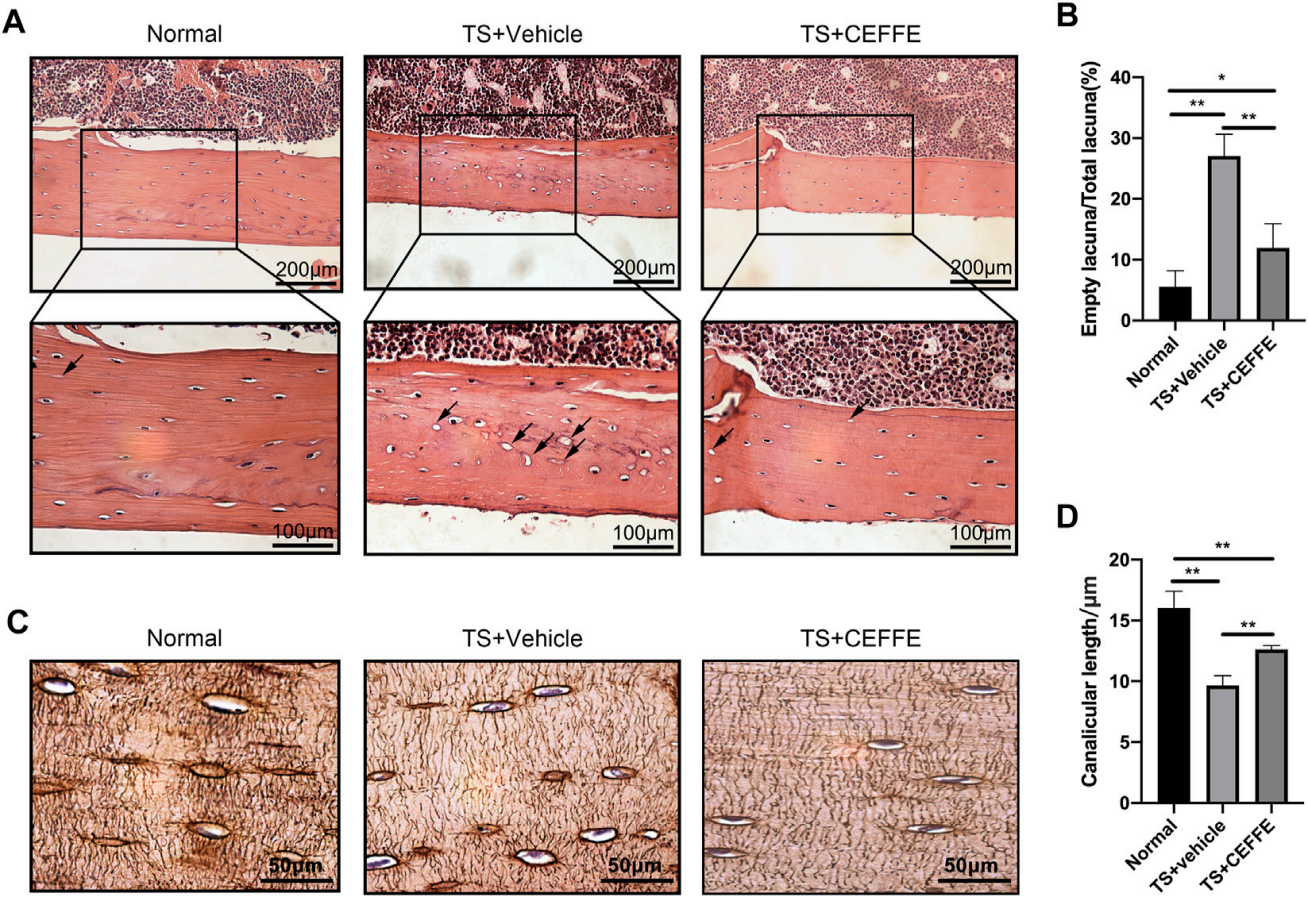
CEFFE improved the lacunocanalicular microstructure in tail-suspended mice. Representative pictures of H&E staining in the femurs **(A)** and quantification of empty bone lacunae **(B)**. Representative pictures of silver staining in the femurs **(C)** and quantification of empty bone lacunae **(D)**. *p < 0.05, **p < 0.01.

H&E staining showed that there were more empty bone lacunas in the cortical bones of tail-suspended mice, which was significantly reduced in the TS + CEFFE group (Figures 2A,B). Tail suspension injured the lengths of the lacunar canaliculi in mice according to silver staining of osteocytes, while the TS + CEFFE group obviously improved the shortening of the lacunocanalicular length caused by tail suspension (Figures 2C,D).

### CEFFE Attenuated Osteocyte Apoptosis and Extracellular Matrix Degradation

**FIGURE 3:**
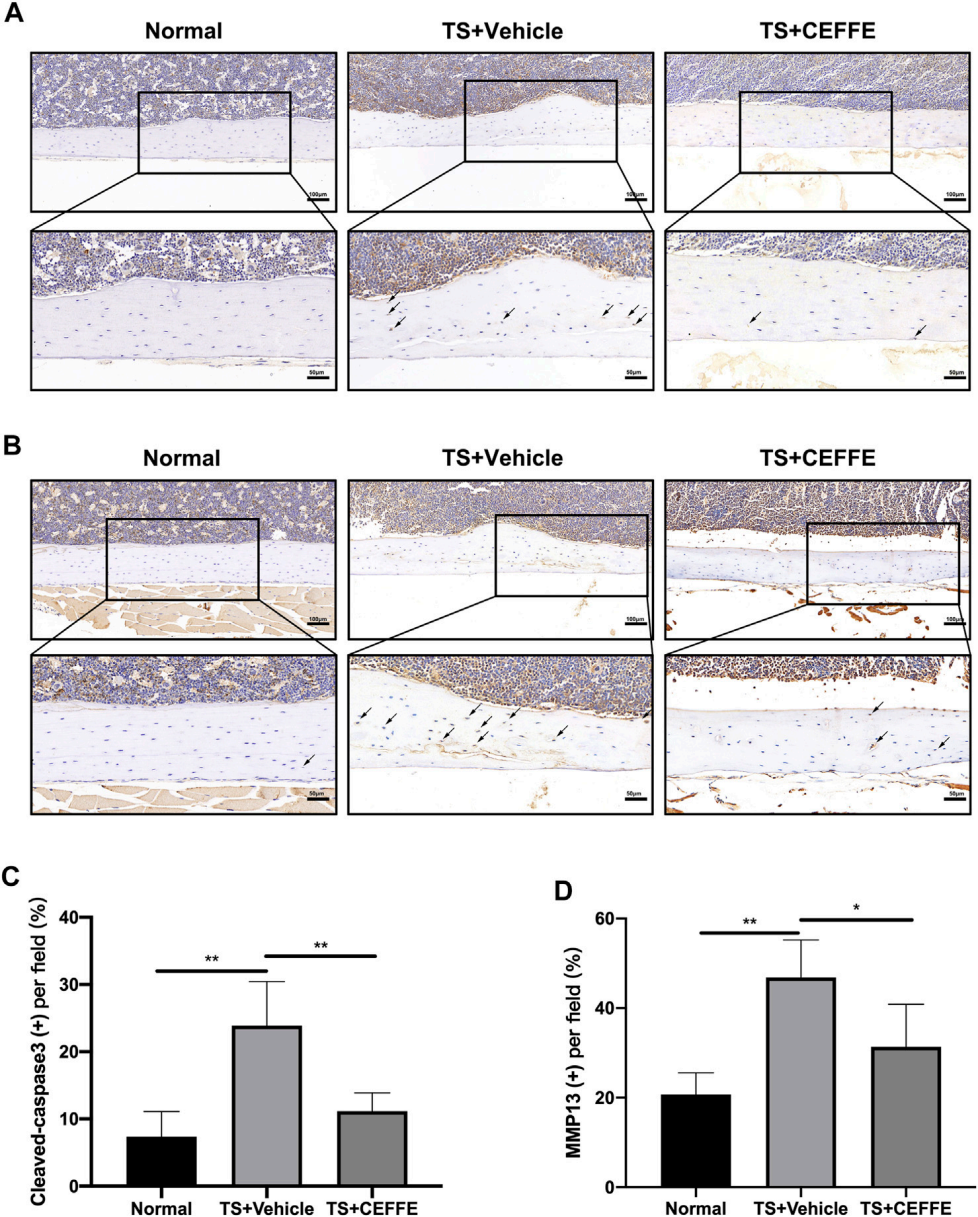
CEFFE attenuated osteocyte apoptosis and extracellular matrix degradation. Immunohistochemical staining and quantitation of femurs shows the expression of cleaved caspase-3 **(A,C)** and MMP-13 **(B,D)**. *p < 0.05, **p < 0.01.

Immunohistochemical staining showed that the TS + CEFFE group had lower levels of cleaved caspase-3 expression in osteocytes than the TS + vehicle group (Figures 3A,B). This is consistent with previous results illustrating that CEFFE resisted osteocyte apoptosis caused by tail suspension. Another surrogate marker of extracellular matrix degradation, MMP13, was also expressed at higher levels in TS + vehicle group mice than in normal mice but not in CEFFE-treated mice (Figures 3C,D). The lower expression of MMP13 may illustrate why the mice in the TS + CEFFE group had a better lacunocanalicular microstructure.

### CEFFE Promotes the Proliferation and Reduces the Expression of MMP13 in Osteocytes *In Vitro*

**FIGURE 4:**
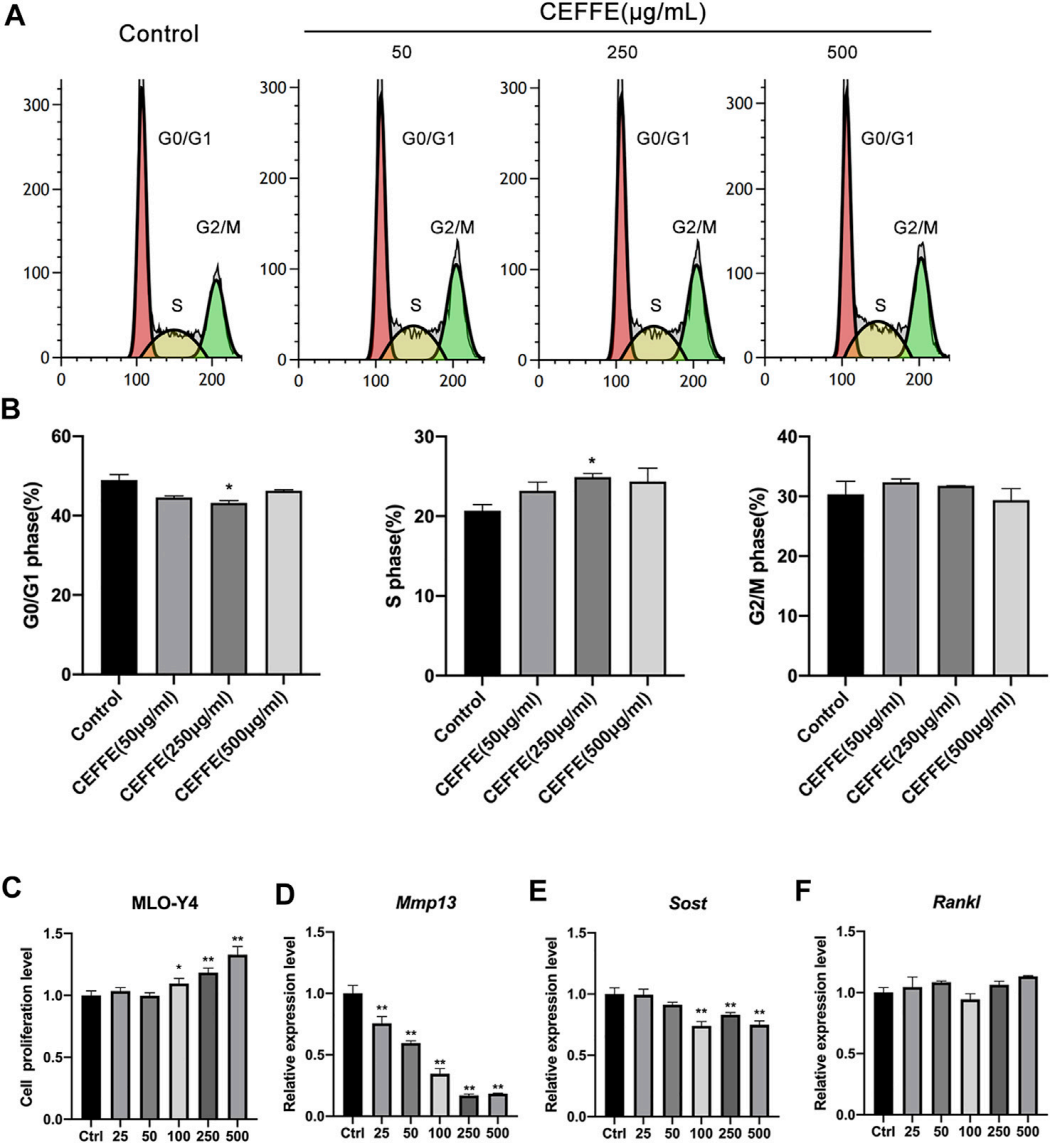
CEFFE promotes the proliferation of osteocytes *in vitro*. Incubation with various concentrations of CEFFE promoted MLO-Y4 proliferation in a dose-dependent manner using flow cytometry **(A,B)** and CCK-8 **(C)**. CEFFE (250 μg/ml) promoted the viability and proliferation of MLO-Y4 cells. Expression of osteocyte-specific and remodeling-related genes, Mmp13 **(D)**, Sost **(E)**, and Rankl **(F)** in MLO-Y4 cells treated with different concentrations of CEFFE for 24 h. *p < 0.05, **p < 0.01 vs. control.

According to the flow cytometry results, CEFFE (250 μg/ml) increased the number of cells in the S phase (Figures 4A,B). Likewise, CEFFE promoted MLO-Y4 proliferation in a dose-dependent manner at concentrations greater than 100 μg/ml (Figure 4C). After incubation with different concentrations of CEFFE for 24 h, MMP13 expressed by MLO-Y4 was significantly decreased in a dose-dependent manner (Figure 4D), and the expression level of SOST was reduced when the concentration of CEFFE was greater than 100 μg/ml (Figure 4E); however, CEFFE culture did not affect RANKL expression (Figure 4F).

### CEFFE Rescued Osteocyte Apoptosis Induced by Reactive Oxygen Species

**FIGURE 5:**
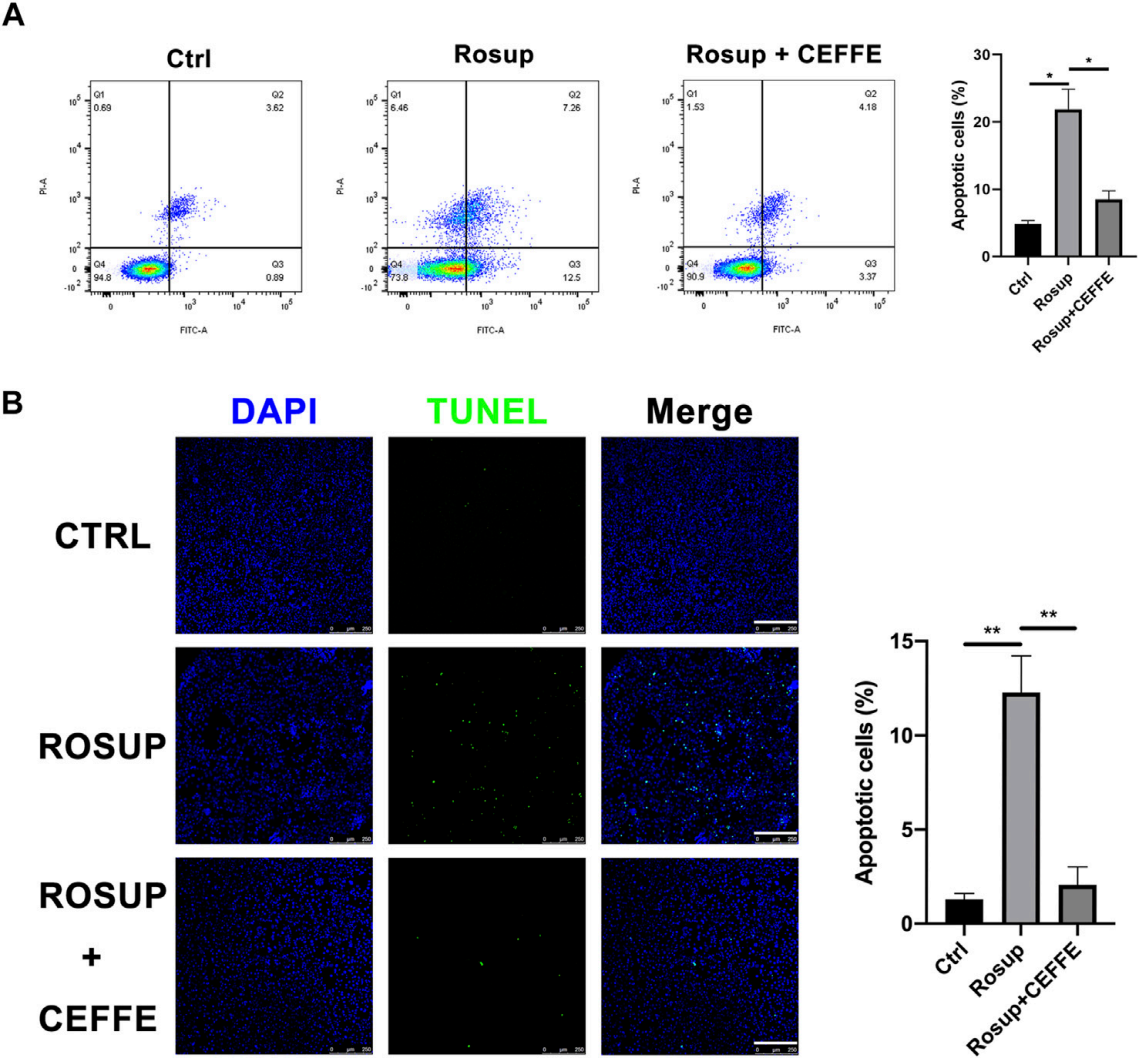
CEFFE rescued osteocyte apoptosis induced by reactive oxygen species. Flow cytometry findings show that CEFFE (250 μg/ml) significantly decreased apoptosis ratios in MLO-Y4 cells induced by Rosup for 8 h **(A)**. Fluorescent green TUNEL staining **(B)**, corresponding blue nuclear counterstaining and merged channels of representative live sections (magnification ×100); ratio (%) of TUNEL-positive cells (N = 3). Scale bars, 200 μm. Data are shown as means ± SD, *p < 0.05, **p < 0.01 vs control.

After treatment with ROS for 8 h, MLO-Y4 showed a marked increase in apoptotic cells according to TUNEL staining and flow cytometry (Figure 5A). When incubated with CEFFE during Rosup administration, the number of apoptotic cells was decreased dramatically, and quantitative differences were detected (Figure 5B).

### CEFFE Inhibited Osteocyte Apoptosis Through the ERK and p38 Signaling Pathways

**FIGURE 6:**
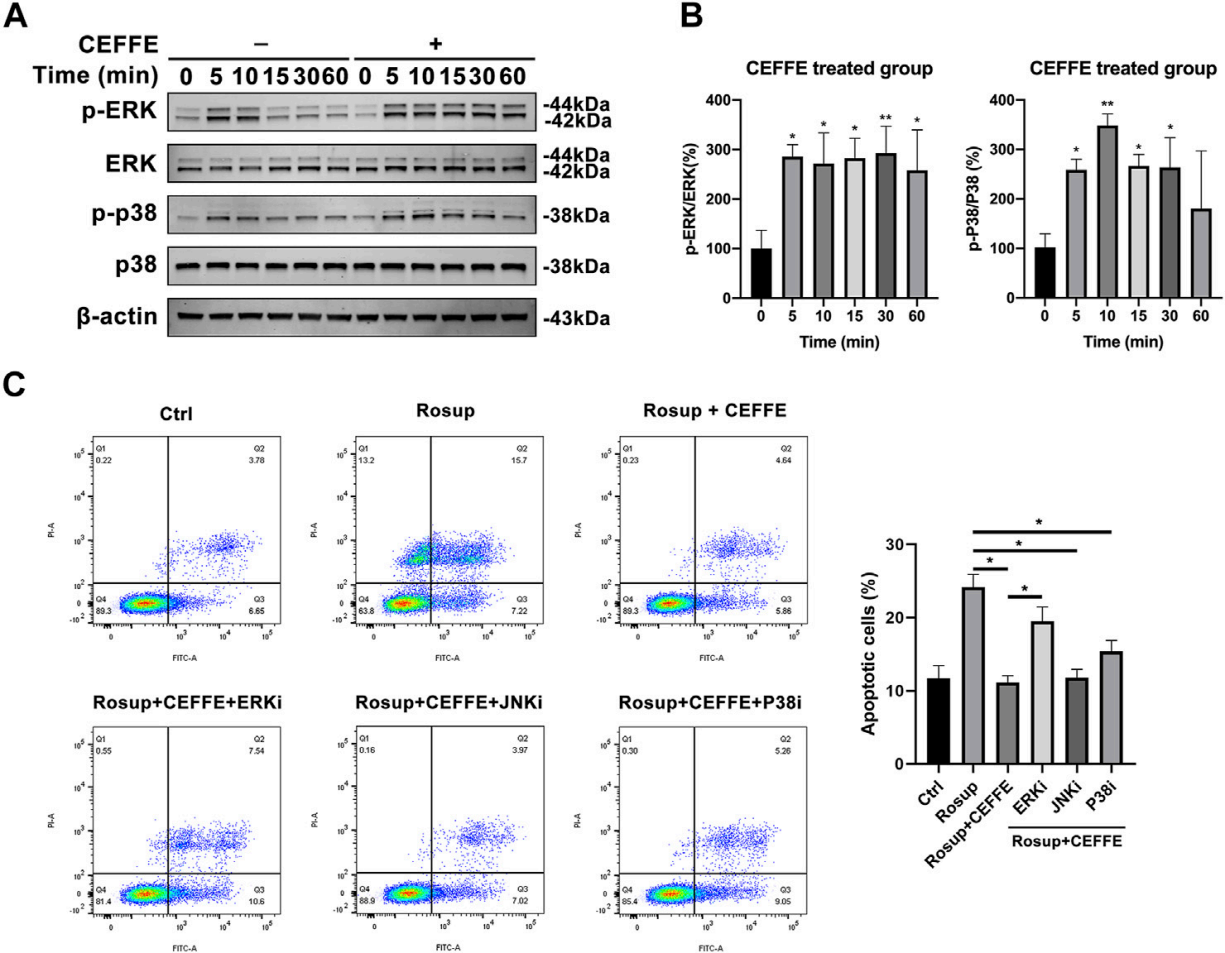
CEFFE inhibited osteocyte apoptosis through ERK and p38 signaling pathway. Cells were incubated with CEFFE (250 μg/ml) for the indicated periods, and proteins were collected within 1 h to assess activation of MAPK signaling pathways, including ERK and p38 **(A,B)**. Incubation with small-molecule inhibitors of MAPK signaling pathways significantly counteracted anti-apoptosis induced by CEFFE **(C)**. *p < 0.05, **p < 0.01 vs. control.

To further explore the antiapoptotic mechanism of CEFFE, Western blotting was applied to assess activation of the MAPK signaling pathway in MLO-Y4 cells. CEFFE significantly induced rapid increases in the phosphorylation of ERK and p38 at 250 μg/ml, which lasted for more than 1 h (Figures 6A,B). Flow cytometry results showed that CEFFE-induced phosphorylation of ERKs was abrogated by treatment of the cells with PD98059 (ERK inhibitors) (Figure 6C).

## Discussion

In the present study, we demonstrated that CEFFE can rescue tail suspension–induced bone loss and recover the lacunocanalicular microstructure. CEFFE was capable of suppressing the pro-apoptosis of MLO-Y4 cells exposed to reactive oxygen species *in vitro*, which was accompanied by an increase in ERK phosphorylation (pERK). Due to the wild source and the effect of anti-bone loss, CEFFE may have potential in treating osteoporosis.

Since CEFFE is extracted from human adipose tissue, many past studies have shown an inverse correlation between adipose tissue content and bone mass. *In vivo*, adipose tissue can affect the growth and development of bone through endocrine pathways. For example, leptin secreted by adipose tissue promotes adipogenesis and reduces osteogenesis in high-fat diet–induced bone-fat imbalance (Yue et al., 2016). This evidence seems to indicate that adipose tissue has a negative effect on bone formation. However, the relationship between bone marrow formation and adipose tissue accumulation in the bone marrow is not always mutually exclusive. Some recent studies have found that adipose tissue plays an indispensable role in maintaining bone mass. For instance, C3H/HeJ mice have both high proximal tibial rBMAT and bone mass (Scheller et al., 2015), although it is difficult for us to clarify the causal relationship between them. Likewise, some newly identified adipocytokines, such as omentin-1, play an essential role in the maintenance of normal bone mass and are able to alleviate magnesium silicate–induced inflammation and osteoporotic bone loss (Rao et al., 2018). Thus, some components in adipose tissue may have a promoting effect on bone mass. Our previous study found that CEFFE is abundant in cytokines, including IGF-1, BDNF, GDNF, TGF-β, HGF, bFGF, VEGF, PDGF, EGF, NT-3, and G-CSF (Yu et al., 2018). These cytokines may affect bone mass; for example, overexpression of IGF-1 upregulated the expression of nuclear β-catenin *via* the AKT pathway, which enhanced cell survival (Lin et al., 2020). Likewise, prior studies validated that VEGF is overexpressed in response to mechanical stimulation and promotes osteocyte survival through a caveolin-1–dependent mechanism (de Castro et al., 2015).

Osteocytes serve as mechanosensitive cells (Li et al., 2021b), and weight loss induces cell apoptosis due to the withdrawal of mechanical stimuli (Basso and Heersche, 2006). These cells have been recognized as multifunctional cells that can regulate osteoclasts through RANKL expression and osteoblasts through sclerostin expression (Robling and Bonewald, 2020); therefore, the death/apoptosis of osteocytes can serve as a signal of resorption and remodeling of bone. According to the immunohistochemistry findings, CEFFE protected osteocytes from apoptosis by reducing the expression of cleaved caspase-3 and MMP13 in cortical bone regions, which indicated that more osteocytes survived and a less number of proteoglycan-rich matrix was degraded (Van Tol et al., 2020). More empty bone lacunae and damaged lacunocanalicular networks (LCN) in unloaded hind limbs of tail-suspended mice were observed, whereas mice treated with CEFFE had more osteocyte survival, and better bone microarchitecture. According to the fluid flow hypothesis, it is difficult for external loading to force bones to deform. Due to the high stiffness of bones, the stress causes the fluid in the LCN to oscillate, and the oscillating fluid flow generates sufficiently strong resistance on the osteocytes to trigger a mechanical reaction (Van Tol et al., 2020). Since mice treated with CEFFE have a more intact LCN structure, osteocytes also have better mechanical sensitivity in this group.

A previous study found that CEFFE significantly upregulates the protein expression of the intracellular antioxidant enzyme glutathione peroxidase-1 and significantly blocks the accumulation of ROS in dermal fibroblasts in UVB-induced cell death (Deng et al., 2019). Yukiko Kitase et al. identified a new function for the muscle-derived metabolite L-BAIBA on osteocyte viability, which protects osteocytes from ROS-induced apoptosis through MRGPRD and through maintaining mitochondrial integrity (Kitase et al., 2018). Therefore, ROS appear to be an intermediate mediator against the protective effects of CEFFE on osteocytes. In a recent study, Rekha Kar et al. found that glucocorticoids activated the MAPK/ERK signaling pathway and increased autophagy and osteocyte survival under oxidative stress (Kar et al., 2019). L. I. Plotkin et al. demonstrated that mechanical stimuli preserve osteocyte viability via activation of the ERK signaling pathway (Plotkin et al., 2005). As a consequence of this finding, we observed that osteocytes treated with CEFFE significantly activated the ERK and p38 signaling pathways. Indeed, rapid phosphorylation of ERK by CEFFE was indispensable for the effects of CEFFE, as their antiapoptotic effects on osteocytes could be almost prevented by a specific inhibitor of ERK activation, GDC-0994. However, CEFFE contains an abundance of growth factors, including IGF-1, TGF-β, VEGF, HGF, bFGF, and many mitogens. Then, we also showed that cell proliferation was significantly increased after 24 h of CEFFE treatment in a dose-dependent manner. Sost is mainly expressed in osteocytes, exhibiting significant inhibition of osteoblast activity and bone formation *in vivo* (Robling and Bonewald, 2020). Hence, the reduction of sost secreted by osteocytes could regulate the survival and differentiation of osteoblasts. We found that incubation with CEFFE could significantly reduce the expression levels of MMP13 and Sost in MLO-Y4 cells, indicating that CEFFE could indeed reduce the degradation of pericellular matrix by osteocytes and reduce the inhibition of the Wnt/β-catenin pathway by Sost to promote anabolism.

Due to the variety of growth factors, a previous study demonstrated that CEFFE was capable of attenuating ischemic injury and stimulating angiogenesis in ischemic tissues (Qin et al., 2019). Therefore, it is likely that the protective effect of CEFFE on osteocytes reported here is also mediated by attenuating limb ischemia from tail suspension or disuse. Based on these observations, we speculate that CEFFE may exert indirect effects on osteocytes by improving the pericellular microenvironment.

There are still many limitations in our study. In this study, although CEFFE treatment rescued bone loss caused by tail suspension, CEFFE treatment did not completely recover the bone mass because of a short-term CEFFE administration. As the treatment time is extended, the increase in bone mass will be more pronounced. Since CEFFE is a mixture rich in various cytokines extracted from adipose tissue, the specific components in CEFFE that have an effect on osteocyte survival are not yet understood, and the specific mechanism remains to be further studied. There are many causes of osteoporosis, in which osteoclasts are hyperactivated in patients with postmenopausal osteoporosis, and whether CEFFE can treat osteoporosis by affecting osteoclast function deserves further study. People may experience bone loss after discontinuation of many osteoporosis drugs, and whether there is a similar discontinuation response after CEFFE treatment requires further study.

## Conclusion

In this study, we showed that CEFFE protected against disuse-induced osteoporosis and that CEFFE increases the survival of osteocytes by activating the ERK pathway. Therefore, CEFFE may be used as a potential drug for the treatment of osteoporosis due to its rich source availability, ease of preparation, and absence of immunogenicity.

## Data Availability

The raw data supporting the conclusion of this article will be made available by the authors, without undue reservation.

## Abbreviations

BMD, bone mineral density; BS/TV, bone surface/total volume; BV/TV, bone volume; CBS, calf bovine serum; CCK-8, Cell Counting Kit-8; CEFFE, cell-free fat extract; Conn.Dens, connection density; ERK, the serine/threonine-selective protein kinase; FBS, fetal bovine serum; H&E, hematoxylin and eosin; MMP13, matrix metalloproteinase-13; LCN, lacunocanalicular networks; OP, osteoporosis; PFA, paraformaldehyde; PVDF, polyvinylidene difluoride; pERK ERK, phosphorylation; ROSUP, reactive oxygen species; Tb.N, trabecular number; qRT-PCR, quantitative real-time PCR; SDS-PAGE SDS‐polyacrylamide gel electrophoresis; TS, tail suspension; μCT, microcomputed tomography; Tb.Th, trabecular thickness; Tb.Sp, trabecular separation; SMI, structure model index.
